# Polyhydroxyalkanoates-Based Nanoparticles as Essential Oil Carriers

**DOI:** 10.3390/polym14010166

**Published:** 2022-01-01

**Authors:** Iolanda Corrado, Rocco Di Girolamo, Carlos Regalado-González, Cinzia Pezzella

**Affiliations:** 1 Department of Chemical Sciences, University of Naples Federico II, Via Cinthia 4, 80126 Napoli, Italy; iolanda.corrado@unina.it (I.C.); rocco.digirolamo@unina.it (R.D.G.); 2 Departamento de Investigación y Posgrado en Alimentos, Facultad de Química, Universidad Autónoma de Querétaro, Cerro de las Campanas s/n, Col. Las Campanas, Queretaro 76010, Mexico; regcarlos@gmail.com; 3 Department of Agricultural Sciences, University of Naples Federico II, Via Università 100, 80055 Portici, Italy

**Keywords:** polyhydroxyalkanoates, nanoencapsulation, in vitro release, antimicrobial activity, essential oil, natural food preservatives

## Abstract

Plant-derived essential oils (EOs) represent a green alternative to conventional antimicrobial agents in food preservation. Due to their volatility and instability, their application is dependent on the development of efficient encapsulation strategies allowing their protection and release control. Encapsulation in Polyhydroxyalkanoate (PHA)-based nanoparticles (NPs) addresses this challenge, providing a biodegradable and biobased material whose delivery properties can be tuned by varying polymer composition. In this work, EO from Mexican oregano was efficiently encapsulated in Polyhydroxybutyrate (PHB) and Poly-3-hydroxybutyrate-co-hydroxyhexanoate (PHB-HHx)-based NPs by solvent evaporation technique achieving high encapsulation efficiency, (>60%) and loading capacity, (about 50%). The obtained NPs displayed a regular distribution with a size range of 150–210 nm. In vitro release studies in food simulant media were fitted with the Korsmeyer–Peppas model, indicating diffusion as the main factor controlling the release. The cumulative release was affected by the polymer composition, possibly related to the more amorphous nature of the copolymer, as confirmed by WAXS and DSC analyses. Both the EO-loaded nanosystems displayed antimicrobial activity against Micrococcus luteus, with PHB-HHx-based NPs being even more effective than the pure EO. The results open the way to the effective exploitation of the developed nanosystems in active packaging.

## 1. Introduction

The growing people awareness for the green consumerism has oriented the food industry towards the use of plant-derived products such as natural and eco-friendly food preservatives [1]. Today, controlling bacterial contamination in fresh or processed foods is a critical issue, especially considering the challenges related to the use of synthetic preservatives, i.e., the possibility of promoting bacterial resistance and their adverse impact on the health and environment. In this scenario, plant essential oils (EOs) are characterized by intrinsic antimicrobial activity against food-borne pathogens and could represent a green and safe alternative to the use of chemical additives. Some EOs and their bioactive compounds, have been already classified as GRAS (Generally Recognized as Safe) by the U.S. Code of Federal Regulations [1]. Furthermore, due to the complexity of their composition, EOs have been shown to exert a multitarget action towards bacteria and fungi, whilst no evidence of occurring resistance towards EOs has been reported [2]. In particular, EO derived from *Origanum* and *Thymus* species, commonly applied in traditional medicine since ancient times, have been shown to exhibit significant antimicrobial activity towards food-borne pathogens, this being property ascribable to their major components, namely the phenolic monoterpene carvacrol and its isomeric form thymol [3,4,5]. Interestingly, this action is less effective towards the beneficial probiotic bacteria *Lactobacillus* [3]. The mechanism behind the observed antimicrobial activity is based on the interaction of these hydrophobic compounds with the bacterial cytoplasmatic membrane that causes its destabilization, increasing its fluidity and permeability for protons and ions, thus determining bacterial cell death [6]. Synergism of such major components with minor ones, including monoterpene hydrocarbons, has been also found to modulate the antimicrobial performance of different populations of *Origanum vulgare* subsp. *hirtum* [7].

Despite such potential, the use of EOs as food preservative still faces some issues related to: (i) the sensitivity to oxygen, light and heat and the reactivity of many extracted compounds; (ii) their limited solubility; (iii) their volatility, that further reduces their availability for bioactivity. Furthermore, EOs can bind to lipids, proteins and carbohydrates present in the food matrixes, therefore requiring higher doses to exert their action, with alteration of the sensory threshold levels [1]. Ultimately, EOs are characterized by intense aroma even at low dose, that may affect the organoleptic properties of the food items.

Encapsulation in nanosystems represents a promising and emerging tool to overcome these challenges. Beside masking their undesirable aroma, loading of EOs into nanomaterials and their controlled release, mostly improve the bioavailability and bioaccessibility of bioactive molecules, by decreasing their volatility, promoting their solubility and stabilization, and optimizing their interaction with food components. Furthermore, with respect to larger reservoirs, nanoencapsulation offers a larger surface area per unit volume, thus possibly increasing the EO concentration at water-rich phases or liquid–solid interfaces, where contaminant microorganisms usually localize inside the food [8].

Considering the lipophilic nature of the EOs compounds, the most effective strategies for their nano-formulation have consisted in the use of lipid-based systems, i.e., nano and microemulsions [9,10], solid-lipid nanoparticles [11], liposomes [12], and polymeric nanoparticles [13,14]. Among the latter category, Polyhydroxyalkanoate (PHA)-based nanoparticles have emerged as biobased, biodegradable and biocompatible materials, potentially applicable in food-packaging [15]. Produced by various species of bacteria trough microbial fermentation of different C-sources (both carbohydrate and lipid ones), PHA origin is properly renewable [16,17]. Furthermore, the diversity in monomeric composition in which they can be produced depending on the microbial bioprocess, makes PHA tunable materials, in terms of mechanical properties and biodegradation kinetics [18]. In recent years, different PHA polymers have been applied for the controlled delivery of various drugs [19,20,21,22]. Furthermore, more recently, the grafting of plain poly-3-hydroxybutyrate-co-hydroxyhexanoate (PHB-HHx) NPs in whey-protein-based film matrix has shown to improve the technological and barrier properties of the functionalized materials, opening the way to the design of smart biomaterials incorporating bioactive molecules inside them [23].

EO from Mexicano oregano (*Lippia graveolens* Kunth) has been previously characterized for its chemical composition, showing thymol as the major compound (66.3%), in addition to γ-terpinene, eugenol, linalool, α-terpineol, and carvacrol [24]. When applied in free form, in nanoemulsions and nanocapsules, it has been found effective towards different food-borne pathogens [5].

The aim of this work is to efficiently encapsulate EO from Mexicano oregano in nanoparticles based on PHB and PHB-HHx copolymer, and to evaluate their oil release kinetics in different food simulants as a function of their different monomeric composition. Furthermore, the antimicrobial activity of these nanosystems was evaluated in comparison with free EO in order to test their potential applicability in food preservation.

## 2. Materials and Methods

### 2.1. Microbial Production of PHA

*Cupriavidus necator* DSM 428 was used for PHB production. The strain was grown aerobically at 30 °C in both rich (Tryptic Soy Broth, TSB) and minimal media (MMCn) according to Budde et al. [25]. For copolymer poly-(3 hydroxybutyrate)-co-(3-Hydroxyhexanoate) (PHB-HHx) production, recombinant *Escherichia coli* strain, LipoB, was cultured in an Eppendorf NewBrunswick, BioFlo/CelliGen^®^ 115 bioreactor according to Corrado et al. [23]. Polymers were recovered according to Corrado et al. [26] and Corrado et al. [23] for PHB and PHB-HHx, respectively.

### 2.2. Essential Oil Extraction

EO was extracted by hydro distillation from *Lippia graveolens*, Mexican oregano (MXO). MXO leaves and flowers were harvested and sun-dried in Toliman (Queretaro, Mexico). A voucher specimen was authenticated and deposited in the Ethno-botanical Collection of the Herbarium of Querétaro “Dr. Jerzy Rzedowski” (QMEX), located at the Faculty of Natural Sciences, University of Querétaro, Mexico (voucher specimen: E. Hernández-Hernández No. 1). Dry material was stored in black polyethylene bags at 25 °C until use. The EO was recovered by hydro-distillation of 400 g of plant material with 5 L of distilled water using a Clevenger-type apparatus (Cristalab, DF, Mexico) [24]. After 3 h, the oily layer on top of the aqueous distillate was removed and dried with anhydrous sodium sulphate obtaining ~15 mL of EO. The EO was stored in sealed vials protected from light at 4 °C until further analysis.

### 2.3. Nanoparticles Preparation via Microemulsification Method

Nanoparticles were prepared according to Corrado et al. [23]. Briefly, two miscible phases were prepared: an aqueous phase and an organic one. The organic phase was prepared dissolving PHB and PHB-HHx in CHCl3 at different concentrations (5, 10 mg mL^−1^). The aqueous phase consisted of 5 mL of sodium dodecyl sulphate (SDS) tested at different concentrations. To obtain EO-loaded nanoparticles, different amounts of EO (0.5–1 mg per mg of polymer) was solubilized in the organic phase before being added to the aqueous one. Nanoparticles were recovered by centrifugation at 5000× *g* for 15 min. The supernatant was used for determining the encapsulation efficiency and loading capacity.

### 2.4. Nanoparticles Characterization

PHA-based NPs were characterized in terms of hydrodynamic size, zeta potential, morphology and encapsulation efficiency.

#### 2.4.1. Particle Size and Z-Potential

NP dispersion (0.1 mg mL^−1^) in water was analyzed for zeta potential and particle size by using a Zetasizer Nano-ZSP (Malvern^®^, Worcestershire, UK). The device was equipped with a helium-neon laser of 4 mW output power with a fixed wavelength of 633 nm (wavelength of laser red emission). The instrument software programmer calculated the zeta potential through the electrophoretic mobility by applying a voltage of 200 mV and using the Henry equation.

#### 2.4.2. Encapsulation Efficiency and Loading Capacity

The encapsulation efficiency percentage (EE %) was determined considering the total phenolic contents of EO. The total phenolic content was estimated using the Folin Ciocalteau reagent as described by Singleton et al. [27]. The calibration curve was plotted by mixing 0.1 mL aliquots of 50, 100, 150, 200, 250, 300 mg mL^−1^ gallic acid solutions with 0.5 mL of Folin Ciocalteau reagent (diluted tenfold) and 0.4 mL of sodium carbonate solution (75 g L^−1^). The absorbance was measured after 30 min at 765 nm. Both EO, and supernatant (containing non-encapsulated EO) upon centrifugation of nanoparticles suspension were subjected to the same assay as described for the calibration curve. The phenolic content of EO was established to be 568 µg per mg of EO.

To determine the encapsulation efficiency, the following equation was employed [13,28]:

(1) $$\mathrm{EE}\;\%=\frac{\left(\mathrm{phenol}\;\mathrm{of}\;\mathrm{EOs}\;\mathrm{used}\right)\mathrm{mg}-\left(\mathrm{phenol}\;\mathrm{in}\;\mathrm{the}\;\mathrm{supernatant}\right)\mathrm{mg}}{\left(\mathrm{phenol}\;\mathrm{of}\;\mathrm{EOs}\;\mathrm{used}\right)\mathrm{mg}}\times 100$$

To determine the loading capacity the following equation was employed:

(2) $$\mathrm{LC}\;\%=\frac{\left(\mathrm{phenol}\;\mathrm{of}\;\mathrm{EOs}\;\mathrm{used}\right)\mathrm{mg}-\left(\mathrm{phenol}\;\mathrm{in}\;\mathrm{the}\;\mathrm{supernatant}\right)\mathrm{mg}}{\left(\mathrm{phenol}\;\mathrm{of}\;\mathrm{EOs}\;\mathrm{used}\right)\mathrm{mg}}\times 100\;$$

### 2.5. In Vitro Release of EO from PHA Based Nanoparticles

In vitro release studies of EO from loaded PHA-based NPs were carried out using a dialysis method according to Chen et al. [28]. A total of 30 mg of NPs was put into a cellulose dialysis tube (Spectra/Por^®^ 3, MWCO, 3.5 kD) containing 1 mL of total volume and transferred into 10 mL of release media and stirred at 150 rpm at 28 °C. The in vitro release was evaluated in distilled water, and in different food simulants: ethanol at 10% (Food Simulant A, FS_A), acetic acid at 3% (Food simulant B, FS_B) and ethanol 20% (Food Simulant C, FS_C) [29]. At regular time intervals, 0.1 mL of supernatant was sucked out and was replaced with an equivalent volume of fresh solution. All EO release experiments were performed in triplicate.

To determine the cumulative release% the following equation was employed:

(3) $$\mathrm{Cumulative}\;\mathrm{release}\%\;\left(t\right)=\frac{C_{t}\times V_{Buffer}+V\sum_{t=0}^{t-1}C_{i}}{\mathrm{EO}}\times 100\;$$

where *C_t_* is the concentration of sample measured at time *t*, *V_Buffer_* is the volume of dialysis buffer *V* is the Volume of sample withdrawn, *C_i_* is the concentration of each sample previous to ‘*t*’, EO is the total amount of the EO encapsulated in the NPs.

### 2.6. Scanning Electron Microscopy (SEM)

A field emission scanning electron microscope (FE-SEM, FEI Nova NanoSEM450) was used to study the morphology of both NPs formulations. A droplet of nanoparticles (suspension in water) was deposited on carbon stickers on aluminum stubs and then dried at room temperature. The images were acquired using an incident electron beam energy between 3 and 5 kV and by collecting secondary electrons (SE) with an ETD or TLD detector.

### 2.7. Thermal and Structural Characterization

The calorimetric thermograms were obtained by scanning with a differential scanning calorimeter Mettler DSC-822 in flowing N_2_ atmosphere and heating and cooling rates of 10 °C/min.

Wide angle X-ray scattering measurements (WAXS) were obtained with Ni-filtered Cu Kα radiation with a Philips automatic diffractometer. Both neat polymer samples and derived loaded NPs were dehydrated under N_2_ flux before the analyses.

### 2.8. Study of Antimicrobial Activity of Loaded PHA Nanoparticles

Minimum inhibitory concentration (MIC) of NPs was determined by a broth dilution method, as recommended by the NCCLS 2000. *Micrococcus luteus* was used as test microorganism [24]. The bacterium was inoculated in NB medium at 37 °C for 24 h. The culture suspension was adjusted to 105 CFU mL^−1^. Bacterial suspension (1 mL) was inoculated in the sample series. After 24 h samples, 10 µL from all tubes without turbidity was transferred to an NB agar plate and incubated at 37 °C for 24 h. The Minimum bactericidal concentration (MBC) was determined as the concentration of the sample which corresponded to no bacterial growth. Tests were performed in triplicate for each sample.

### 2.9. Statistical Analyses

Analyses were conducted using Minitab 17 Statistical Software (2010). Experiments were conducted in triplicate and arithmetic means and mean square errors were calculated. Significant differences in average values were assessed using the Tukey–Kramer HSD test (significance level: *p* < 0.05).

## 3. Results

### 3.1. Encapsulation of Essential Oils in PHA-Based Nanoparticles

#### 3.1.1. PHB-Based Nanoparticles

PHA-based nanoparticles were prepared by the solvent evaporation method, by dispersing a polymer solution in the aqueous phase containing a proper surfactant. Two different PHA polymers were tested: (i) the homopolymer Polyhydroxybutyrate (PHB), and (ii) the copolymer poly-(3 hydroxybutyrate)-co-(3-Hydroxyhexanoate) (PHB-HHx).

Oregano essential oil (EO) was extracted from *Lippia graveolens*, Mexican oregano (MXO) by hydro-distillation, obtaining a yield of 4% (*w/w* dry mass). Besides other minor components, these EOs, already characterized by GC-MS, are mainly composed of thymol (>50%) whilst are characterized by a low concentration of Carvacrol (<0.1%) [24].

Encapsulation of EO into PHA nanoparticles was carried out by exploring, sequentially, the effect of different parameters, such as concentration of surfactant (2.2 and 4.4 mg mL^−1^), Aqueous/Organic phase volume ratio (A/O phase) (20, 8, 2) and mg_EOs_/mg_Polymer_ ratios (1:1; 0.5:1).

Table 1 reports the mean particle size, polydispersity index (PDI) and Z-potential of NPs obtained in the experiments carried out using PHB polymer.

Trials S1–S6 explore the effect of SDS concentration, and mg_EOs_/mg_Polymer_ ratio, at a constant A/O phase (20), the latter corresponding to 1.25 mg of polymer. Data from trials S1 and S2 indicate that in the absence of EO, SDS at 4.4 mg mL^−1^ provided the formation of smaller particles (below 150 nm) with a Z-potential below −60 mV and a PDI of 0.23, while a decrease of SDS concentration led to higher particle size and PDI index, while preserving a good stability (Trials S1, S2). The addition of the essential oils in the organic phase at the two tested ratios caused a marked increase in NP size, in all the tested conditions (Trials S3–S6). Moreover, an increase in PDI was also observed, indicating a higher heterogeneity in the particle size distribution (Figure S1). It is worth to note that the formation of EO micelles with a size of 500 nm was observed when EO were subjected to the same experimental protocol in the absence of polymer. Thus, the formation of SDS-stabilized EO emulsions could explain the observed heterogeneity of particle size, especially in the conditions with higher SDS amount (Trial S5). In line with this, at a lower mg_EO_/mg_Polymer_ (0.5), the PDI decreases to about 0.4, since a greater amount of polymer is available for the interaction with the EO, thus preventing the formation of more homogeneous EO micelles (Trial S4 and S6).

In order to promote polymer/EO interaction and thus improving oil encapsulation into the polymeric matrix, the effect of lowering the A/O phase ratio (8, 2), corresponding to an improvement of polymer amount (3.125 and 12.5 mg, respectively), was tested, setting the SDS at 2.2 mg mL^−1^ (Trials S7–S10). The latter, being below the critical micellar concentration for SDS (2.36 mg mL^−1^) would also discourage the formation of self-assembling SDS micelles.

When the A/O phase ratio was progressively reduced, a significative reduction in particle size was observed in the absence of EO (Trials S7, S9). More interestingly, monodisperse, and very stable NPs were achieved (Figure S2). Furthermore, the addition of EO slightly affected the size of the NPs when compared to the unloaded ones and did not impair the PDI that remains about 0.2 (Trials S8, S10). The decrease in the A/O phase ratio, i.e., reducing the volume of the organic phase, allows to reduce the occurrence of coalescence phenomenon because the droplets of dispersed phase (chloroform) are present at a greater distance in the continuous phase (water), attenuating their aggregation [30,31,32]. The observed improvement in NPs polydispersity and their size reduction, can be also explained considering the effect of the higher polymer availability in the emulsion. The latter promoted the interaction polymer/EO with respect to the formation of SDS-stabilized EO micelles.

In the following experimental set, the effect of a further increase of polymer amount was tested, by keeping the A/O phase ratio to 2 while increasing the polymer concentration to 10 mg mL^−1^ (corresponding to 25 mg of polymer) (Trials S12–S14).

In these conditions, an increase in the size of unloaded NPs was observed (S12). The increase in particle size is also associated with a slight reduction in the Z-potential (compare trials S9/S12 and S10/S13). This charge reduction may be due to a lower surface coverage by the SDS, since the same amount of SDS had to cover larger NPs. The addition of EO did not significantly affect the particle size at the highest mg_EO_/mg_Polymer_ (S13). Conversely, particle size was reduced in the presence of a lower amount of EO (S14), probably because of a lower EO loading per each nanoparticle. Furthermore, the PDI was almost unaffected by the addition of the EO. As a fact, very monodisperse NPs were obtained after EO encapsulation, indicating that in these conditions, the synthesis of loaded polymeric NPs is favored with respect to the formation of EO micelles.

**Figure 1:**
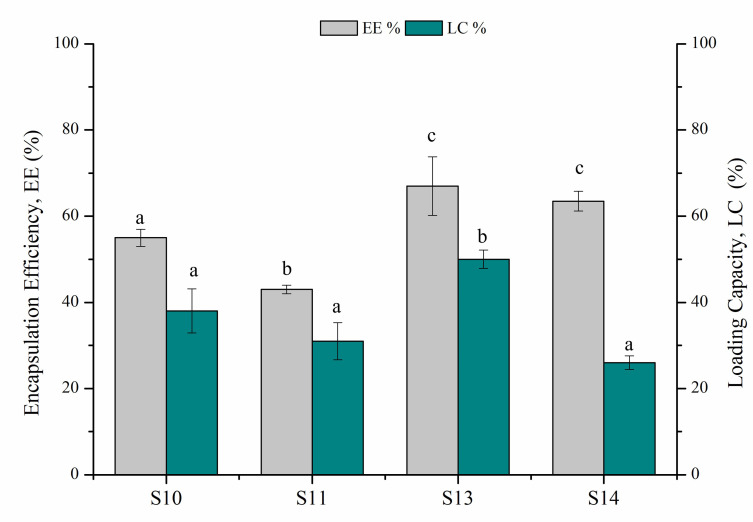
Effect of PHB polymer concentration and mg_EO_/mg_Polymer_ ratio (*w/w*) on Encapsulation efficiency, EE%, and Loading capacity, LC%. S10, S11, S13 and S14 correspond to samples reported in Table 1 (Means that do not share a letter are significantly different).

The conditions S10, S11, S13, S14 were then further investigated, taking into account the EO encapsulation efficiency (EE) and the loading capacity (LC) (Figure 1).

Both the maximum EE% and LC% were obtained at the highest amount of polymer (10 mg mL^−1^), reaching up to 70% and 50%, respectively, at the highest mg_EO_/mg_Polymer_ ratio (S13). A comparable EE was obtained by reducing the mg_EO_/mg_Polymer_ (S14), although a lower LC was observed, in accordance with the lower NP size measured for this sample.

In all the tested conditions the loaded NPs resulted to be stable for more than one week without separation, precipitation or agglomeration.

#### 3.1.2. PHB-HHx Based Nanoparticles

The same experimental conditions used for PHB were applied for the synthesis of PHB-HHx-based NPs (Table 2, Trial H1-H2-H3).

**Figure 2:**
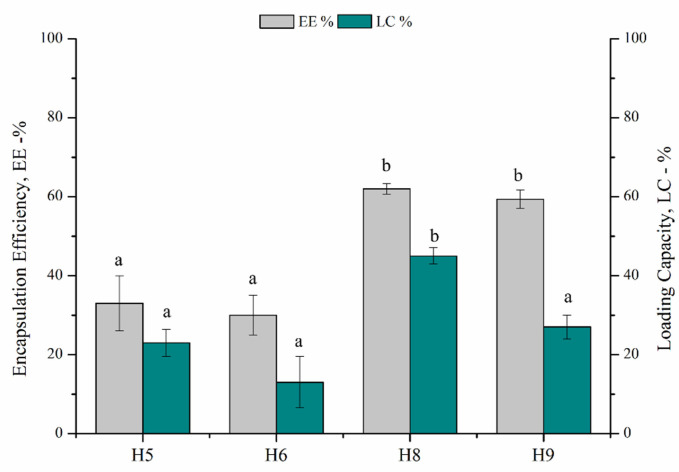
Effect of PHB-HHx polymer concentration and mg_EO_/mg_Polymer_ ratio (*w/w*) on Encapsulation efficiency, EE%, and Loading capacity, LC%. H5, H6, H8 and H9 correspond to samples reported in Table 2 (means that do not share a letter are significantly different).

However, despite the obtained NPs showing a particle size of 180 nm with a PDI of 0.176 and an acceptable Z- potential, they resulted to be unstable, showing precipitation and phase separation after 48 h. To obtain monodisperse and stable NPs, a higher concentration of surfactant (4.4 mg mL^−1^) was needed; moreover, to promote polymer/EO interaction and thus improving oil encapsulation into the polymeric matrix, a higher concentration of polymer solution (20 mg mL^−1^) was used (Table 2 and Figure 2).

The encapsulation of EO slightly affected the particle size with respect to the unloaded ones at lower polymer concentration (Trials H2–H3 and H5–H6). By increasing polymer concentration, a more marked increase in NPs dimension was achieved, in accordance with the higher EE% measured for these samples (Figure 2).

The highest EE and LC values were obtained in H8, corresponding to the synthesis of stable and monodisperse NPs with a particle size of 210 nm (Figure 2). In general, at higher concentration of polymer, the collision frequency of polymer droplets and possible coalescence can increase. In this case this phenomenon is avoided by using higher concentration of surfactant that helps the formation of droplets and reduces coalescence [33], determining the formation of high-loading and monodisperse NPs.

In conclusion, the Encapsulation efficiency, EE% (>60%) and Loading capacity, LC% (about 50%) obtained for both PHB and PHB-HHx-based nanosystems, are among the highest found for EO encapsulation in different nano-systems: EE% ranging from 5.4% to 24.72% was observed for encapsulation of Oregano EO in chitosan nanoparticles [34], whilst Thyme EO loaded in chitosan nanoparticles showed about 30% EE [35]. An improved EE% (>95%) has been reported by Granata et al. [14] for encapsulation of Oregano and Thyme EO in polymeric poly(ε-caprolactone) nanocapsules, together with about 55% Loading capacity.

### 3.2. Morphological, Structural and Thermal Analyses of PHB and PHB-HHx NPs

**Figure 3:**
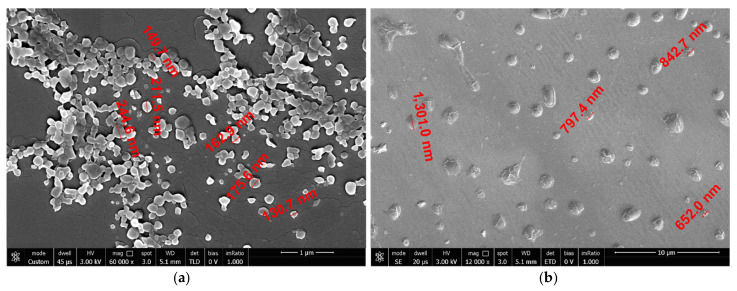
Scanning electron microscopy (SEM) images of EO-loaded NPs. PHB NPs (**a**); PHB-HHx NPs (panel **b**). The size of both nanoparticles (**a**) and aggregates (**b**) are indicated in red.

EO-loaded NPs derived from conditions S13 and H8 were analyzed by Scanning Electron Microscopy (Figure 3). The PHB-loaded nanoparticles were found to be spherical in shape even if with an irregular surface (Figure 3a). The loss of perfect sphericity has been probably caused by the drying process, necessary to conduct SEM characterization. The size of the particles is quite homogeneous and in accordance with data from Z-seizer. The behavior is different for PHB-HHx-loaded NPs, as a fact upon drying the NPs lost their spherical morphology and this can be due to the more flexible nature of this copolymer (Figure 3b).

**Figure 4:**
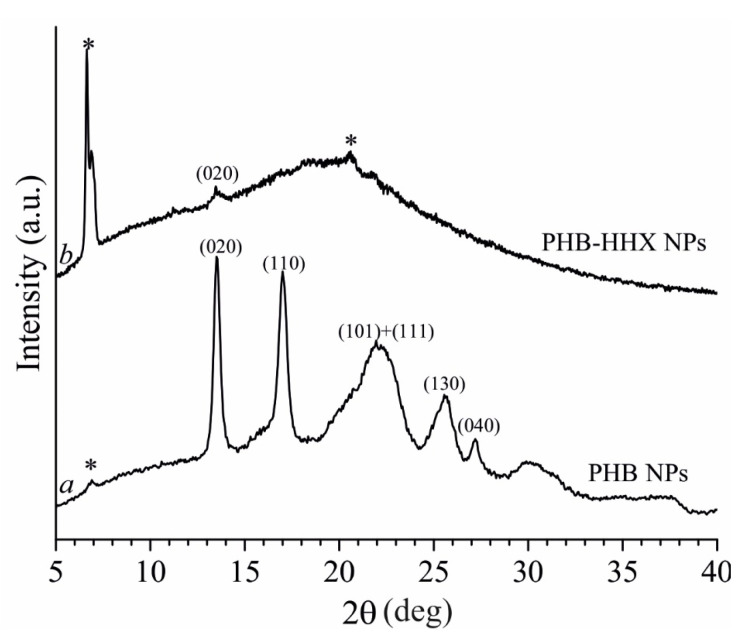
X-ray powder diffraction profiles of the EO-loaded nanoparticles PHB NPs (**profile a**) and PHB-HHx NPs (**profile b**). The diffraction of residual SDS crystals is marked (*).

The crystalline or amorphous nature of PHB and PHB-HHx nanoparticles was directly probed by performing Wide Angle X-ray measurements (WAXS) on dehydrated aliquots of nanoparticles loaded with EO. As shown by the diffraction profiles reported in Figure 4, the PHB NP sample is highly crystalline, according with the presence of reflections (020), (110), (101) and (111) of PHB α form located at around 2θ = 13°, 17°, 21.5° and 22.5°, respectively [36].

In the case of the PHB-HHx sample, the typical broad halo of the amorphous is observed in the diffraction profile b of Figure 4 along with the presence of (020) reflection at 2θ = 13° with very low intensity indicating that only traces of crystallinity are detected in this sample, probably due to the PHB regular sequences present in the copolymer backbone. It is worth noting that in both diffraction profiles, extra diffraction peaks (indicated with an asterisk) due to the diffraction of residual SDS crystals present in the sample are visible.

We are aware that the structure observed by WAXS analysis in dehydrated nanoparticles might differ from that one present in solution, but we are also confident that the crystallinity is not strongly influenced by the dehydration process.

X-ray powder diffraction profiles collected on neat samples, as obtained from microbial production, (Figure S3) are similar to those of loaded NPs discussed above, confirming the presence of traces of crystallinity for the copolymer PHB-HHx.

**Figure 5:**
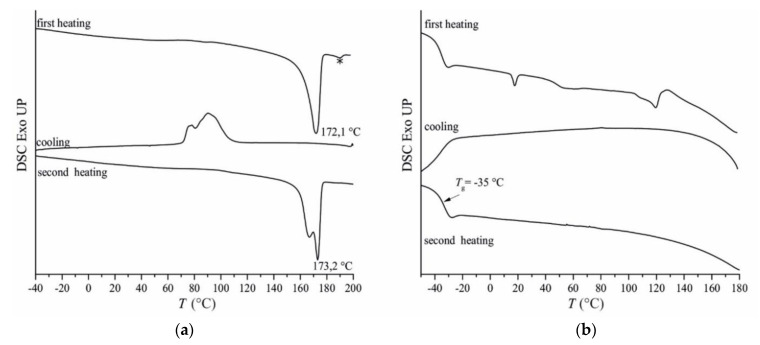
DSC thermograms, acquired at 10 °C/min of the EO-loaded nanoparticles PHB NPs (**a**) and PHB-HHx NPs (**b**). The melting/crystallization of residual SDS crystals is marked (*).

Differential scanning calorimetry (DSC) curves of dehydrated nanoparticles recorded during heating, successive cooling, and second heating are shown in Figure 5.

The DSC thermogram of PHB NPs sample presents a melting peak at 172.1 °C (first heating scan), a crystallization peak at 92 °C (cooling scan) and again the melting event is observed in the second heating thermogram (Figure 5a). The peak observed at temperature higher than 180 °C is due to the melting/crystallization of residual SDS crystals (marked with and asterisk). For the sample PHB-HHx, the first heating scan (Figure 5b) reveals a glass transition temperature at −35 °C followed by multiple and broad endotherms with weak intensity between 0 and 100 °C due to the presence of polymer crystalline sequences and/or to the associated evaporation of volatile oil fractions and solvent during heating. DSC curves recorded during cooling and second heating do not show crystallization and melting phenomena.

DSC thermograms were collected also for neat PHB and PHB-HHx samples (Figure S4). Whilst in the case of neat PHB and PHB nanoparticles with EO DSC thermograms are similar, it is worth highlighting that for the sample PHB-HHx an evident decrease of the glass transition temperature is observed for the EO-loaded PHB-HHx-NPs sample, −35 °C compared with the *T*_g_ = −7 °C of the neat PHB-HHx sample (Figure S4). This is a clear indication that the essential oils penetrate in the amorphous phase and act as plasticizers. A similar behavior has not been observed in the case of PHB probably because of the crystallinity associated with this system.

Both thermal and structural characterizations highlight the difference in crystallinity present in the studied samples that would possibly affect the release properties of EO-loaded NPs.

### 3.3. In Vitro Analysis of EO Release from PHB and PHB-HHx Loaded NPs

The release profile was studied for EO loaded PHB and PHB-HHx NPs, produced under conditions S13 (PHB) and H8 (PHB-HHx), characterized by 68% and 62% of EE and 51% and 45% of LC, respectively.

**Figure 6:**
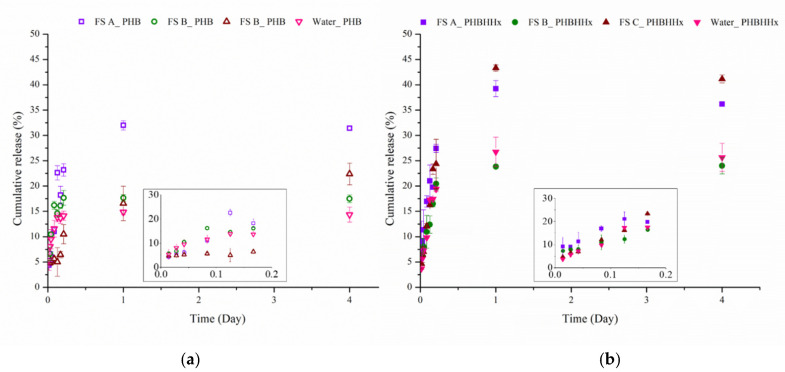
Cumulative release profile of EO-loaded PHB (**a**) and PHB-HHx (**b**)-based NPs in different aqueous food stimulants. FS_A, square; FS_B, pointing up triangle; FS_C, circle; Water, pointing down triangle. Empty points correspond to PHB-based NPs, Full coloured points to PHB-HHX based ones. Release phase in the earliest time is highlighted in the inset plots.

The release was studied by dialysis method in water and in different aqueous food simulants, according to the Commission Regulation (EU) No 10/2011 (Directive 2002/72/EC), a specific measure for plastic food contact materials [29]. In all the tested conditions, the release profile is biphasic for both NPs, showing an initial burst in the first 6 h, followed by a constant release during the time until stabilization (Figure 6).

The maximum release in a 4-day window in all the food simulants was always higher for the copolymer with respect to the homopolymer, fairly related to the more amorphous character of the PHB-HHx. In particular, the release was the highest in food simulant C, with around 40% after 4 days for PHB-HHx and 30% for PHB. The presence of 20% Ethanol also seems to affect the release kinetics in the first part of the curve, amplifying the difference between the polymers. Comparing the behavior of PHB and PHB-HHx-based NPs in food Simulant A and B, the maximum release achieved after 4 days was 29% vs. 35% and 17% vs. 23%, respectively, whilst the differences in the burst release region were less pronounced.

The same trend was also found for EO release in distilled water, with PHB NPs achieving a maximum release of 15% after 4 days versus 26% for the copolymer.

A modification of the Korsmeyer–Peppas mathematical model [37] was used to investigate the EO release from PHA nanoparticles. This model is used to fit the initial 60% of the release and include the rapid release of drug in the first time of incubation, according to the following equation [38]:

(4) $$\frac{M_{t}}{M_{\infty }}=K_{1}t^{n}+b$$

where *M_t_* amount of drug release after time, *t*, *M*_∞_ = amount of drug release at infinite time, or total drug encapsulated, *K*_1_ = release constant as function of time, *n* = release exponent, *t* = time and *b* = burst effect.

The EO release in the all the different media showed a good fit, with a R^2^ above 0.86 (Table 3).

Exponent *n* gives information about the mechanism of release, for *n* ≤ 0.43 the release is a Fickian diffusion while for *n* = 0.85 it is a case transport II, related to polymer matrix relaxation and swelling. Moreover, if 0.43 < *n* < 0.85 the diffusion corresponds to an anomalous transport, as a result of both mechanisms [39]. For EO release from PHB-based NPs, *n* values in all conditions, except for Food Simulant C, turned out to be lower than 0.43, indicating the predominant release of drug due to Fickian diffusion. The EO release in the food simulant C showed a *n* value higher than 0.43, indicating an anomalous transport, probably caused by an effect of the solvent on the structure of the polymer matrix. For the release from PHB-HHx-based nanoparticles the Fickian diffusion prevailed in all tested conditions.

According to the model, the rate of release depends on release medium. For PHB-based nanoparticles in food simulant A the rate was faster than the other studied systems (K = 19.96), whilst the food simulant B and C showed a comparable release rate (11.97 vs. 10.58). On the other hand, for PHB-HHx-based NPs, the release rate was in the following order Simulant C > Simulant A > Simulant B. It is worth to note that the release rate in food simulants A and B is almost comparable between the two nanosystems, whilst the high ethanol concentration in food simulant C determines a significant increment of the release rate for the copolymer (10.58 vs. 25.58). As far as the release kinetics in distilled water, the EO release rate from PHB NPs was significantly lower than PHB-HHx ones, confirming that the water access in the more amorphous matrix of the copolymer is more favored, thus boosting the release.

The release profiles are influenced by several factors such as temperature, pH, viscosity of the solution, kind of encapsulated molecule, dimension of NPs and type of polymer [40,41]. Several authors have investigated the release of essential oils encapsulated in different kinds of polymeric matrixes. Hosseini et al. reported a range 12–82% cumulative release of Oregano EO encapsulated in chitosan NPs after 3 h, followed by a slow release, depending on EO loading and NPs dimension [34]. Similarly, a burst release (20–30% in the first 8 h) followed by a slower release rate (60% after 7 days) has been observed for docetaxel-loaded P(3HB-co-4HB) nanoparticles with low drug/polymer ratio, whilst a higher ratio led to a relatively rapid release (almost complete after 48 h) [42]. Granata et al. [14] outlined the effect of the essential oil type in the kinetics of release from poly(ε-caprolactone)-based NPs, although reporting a very slow-release behavior (maximum 10–15% release at 40 °C). When a mathematical model has been applied to describe the release kinetics of a lipophilic probe from poly(R-3-hydroxybutyrate-co-1,4-butylene adipate) copolymers, the best fitting corresponded to the Korsmeyer–Peppas model although, differently from our findings, diffusion and erosion have been both supposed to be involved in drug leakage [43].

Our results indicate that the synthesized NPs can be considered promising candidates for prolonged delivery use, assuring the slow release of EO over time in all the tested food simulants, thus being suitable for developing active packaging applications. The possibility to modulate the release kinetics by exploiting PHB- or PHB-HHx-based NPs, could be useful to address their use to prolong the shelf life of foods requiring different storage times.

### 3.4. Assessment of Antimicrobial Activity of EO Loaded Nanoparticles

The antimicrobial activity of PHA-NPs loaded with EO was investigated determining the Minimum inhibitory concentration (MIC) and the minimum bactericidal concentration (MBC) against Micrococcus luteus NCIB 8166. The latter was chosen as test strain because of its high sensitivity to the tested EO [5]. Samples derived from trials S13 and H8 were evaluated in comparison with free EO (Table 4).

Both loaded nanoparticles were found to be effective against M. luteus. In particular, for PHB-based nanoparticles, no turbidity occurred when 0.5 mg mL^−1^ of NPs was added. A lower MIC was determined for the copolymer-based nanoparticles, corresponding to 0.25 mg mL^−1^. The same trend was also observed for the MBC, which was higher for PHB-loaded NPs with respect to PHB-HHx-based ones. Unloaded nanoparticles did not affect microbial growth up to 2 mg mL^−1^ (data not shown).

It is worth to note that encapsulated EO possesses high inhibitory and bactericidal activity, being the copolymer-based NPs even more effective than the pure EO (Table 4). As a fact, both the MIC and MBC of free EO against M. luteus were reduced from 0.2 to 0.11 mg mL^−1^ and from 0.3 to 0.18 mg mL^−1^, respectively, by PHB-HHx-based NPs. This enhanced antimicrobial activity is imputable to their nanoencapsulation that can not only protect the active compounds from oxidation or other environmental factors (water, light, oxygen, pH), but also promote their distribution and solubility through controlled release, thus enhancing their bioavailability [1]. A similar effect was observed for EO from *Origanum dictamnus* L. encapsulated in liposomes [44], and for *Thymus capitatus* and *Origanum vulgare* EO encapsulated in poly(ɛ-caprolactone) (PCL) nanocapsules [14], although against different test organisms. An enhanced antimicrobial effect has been verified also for purified bioactive compounds, i.e., carvacrol. When encapsulated in nano-emulsions, it showed significantly enhanced diffusivity resulting in a more efficient antimicrobial action [8]. In contrast, other authors reported examples in which encapsulating EO or purified bioactive molecules into different nano-systems has been not advantageous in terms of antimicrobial effect with respect to the free counterpart, this behavior being mainly ascribable to the rate of antimicrobial release, which in turn is dependent on the type of encapsulating agent [5,13].

Although differences in the applied methodologies and test microorganisms prevent the comparison with previous reports on the antimicrobial effect of encapsulated Mexican oregano EO, this work represents the first example of exploiting PHA-based nanoparticles for the delivery of essential oils. Interestingly, the observed differences in bacteriostatic and bactericidal behavior of the two kinds of tested NPs, ascribable to their different rates of oil release, point out the flexibility of this kind of polymer, that may represent a tuneable and useful polymer matrix to design proper application in food packaging.

## 4. Conclusions

Oregano essential oil was efficiently encapsulated into PHA-based nanoparticles by means of solvent evaporation technique. High encapsulation efficiency and Loading capacity were achieved optimizing the process parameters for both PHB and PHB-HHx polymers.

Besides, the prepared NPs showed a monomodal size distribution (PDI ≤ 0.16). The release curves of the EO from the two polymeric matrixes in different conditions was found to fit the Korsmeyer–Peppas mathematical model, being diffusion, the main mechanism controlling the release. The cumulative release was the highest in the case of the copolymer, possibly related to its more amorphous nature, as confirmed by WAXD analysis.

EO encapsulation into both nanosystems translated into high inhibitory and bactericidal activity against M. luteus, the PHB-HHx-based NPs being even more effective than the pure EO.

Taken together, the results encourage further investigation about the effective application of the synthesized nanosystems in the functionalization of packaging materials for the long-term preservation of food from microbial contamination. Besides adding useful activities (antimicrobial, antioxidant etc.) to functionalized materials, the addition of nanosystems could also affect the technological features of the materials (i.e., permeability to water and gases, mechanical properties), thus opening the way to the design of improved nanocomposites.

## Figures and Tables

**Table 1:** Effect of different parameters on the synthesis of PHB-based nanoparticles. A/O, Aqueous/Organic phase volume ratio, PDI, polydispersity index, d., diameter. Values are mean ± SD; the different letters indicate significant differences from the values reported in the same column (Tukey-Kramer test, *p* < 0.05). The different letters indicate significant differences.

Trial	SDS (mg mL^−1^)	PHB (mg mL^−1^)	A/O Phase	mg_EO_/mg_PHB_	d. (nm)	PDI	Z-Pot (mV)
S1	2.2	5	20	-	172.4 ± 8.6 ^a,b^	0.29 ± 0.04 ^a,b^	−40.1 ± 0.9 ^c,d,e^
S2	4.44	5	20	-	115.3 ± 10.0 ^a^	0.23 ± 0.03 ^a^	−62.2 ± 8.5 ^a^
S3	2.2	5	20	1	459.8 ± 51.1 ^a,b^	0.62 ± 0.21 ^b^	−18.2 ± 0.4 ^f^
S4	2.2	5	20	0.5	248.3 ± 51.1 ^a,b^	0.49 ± 0.21 ^b^	−16.5 ± 0.4 ^f^
S5	4.44	5	20	1	541.6 ± 383.9 ^c^	0.80 ± 0.17 ^c^	−18.7 ± 8.5 ^f^
S6	4.44	5	20	0.5	357.4 ± 68.3 ^b,c^	0.46 ± 0.06 ^b^	−18.2 ± 4.4 ^f^
S7	2.2	5	8	-	120.7 ± 2.8 ^a^	0.41 ± 0.11 ^b^	−35.1 ± 0.2 ^e^
S8	2.2	5	8	1	127.3 ± 1.1 ^a^	0.23 ± 0.03 ^a^	−36.1 ± 1.4 ^d,e^
S9	2.2	5	2	-	112.7 ± 1.3 ^a^	0.13 ± 0.01 ^a^	−45.5 ± 7.6 ^c,d^
S10	2.2	5	2	1	128.0 ± 5.8 ^a^	0.15 ± 0.03 ^a^	−50.6 ± 3.5 ^b,c^
S11	2.2	5	2	0.5	141.4 ± 2.6 ^a^	0.17 ± 0.02 ^a^	−55.0 ± 2.6 ^a,b^
S12	2.2	10	2	-	152.8 ± 6.8 ^a^	0.19 ± 0.04 ^a^	−41.4 ± 1.2 ^d,e^
S13	2.2	10	2	1	156.4 ± 1.7 ^a^	0.16 ± 0.01 ^a^	−44.9 ± 3.7 ^d,e^
S14	2.2	10	2	0.5	113.1 ± 1.7 ^a^	0.12 ± 0.02 ^a^	−37.1 ± 2.4 ^c,d^

**Table 2:** Effect of different parameters on the synthesis of PHB-HHx-based nanoparticles. A/O, Aqueous/Organic phase volume ratio, PDI, polydispersity index, d., diameter. Values are mean ± SD; the different letters indicate significant differences from the values reported in the same column (Tukey–Kramer test, *p* < 0.05). The different letters indicate significant differences.

Trial	SDS (mg mL^−1^)	PHB-HHx (mg mL^−1^)	A/O Phase	mg_EO_/mg_PHBHHx_	d. (nm)	PDI	Z-Pot (mV)
H1	2.2	10	2	-	174.6 ± 2.2 ^a,b^	0.09 ± 0.03 ^a^	−35.5 ± 0.3 ^a^
H2	2.2	10	2	1	180.0 ± 11.0 ^a,b^	0.18 ± 0.01 ^b^	−40.4 ± 2.2 ^a,b,c^
H3	2.2	10	2	0.5	196.4 ± 18.1 ^b,c^	0.24 ± 0.01 ^c^	−45.7 ± 5.4 ^c,d^
H4	4.4	10	2	-	166.6 ± 2.1 ^a^	0.09 ± 0.01 ^a^	−38.2 ± 0.9 ^a,b^
H5	4.4	10	2	1	174.2 ± 1.2 ^a^	0.12 ± 0.02 ^a,b^	−44.2 ± 1.2 ^b,c,d^
H6	4.4	10	2	0.5	175.3 ± 3.3 ^a,b^	0.14 ± 0.02 ^a,b^	−41.2 ± 0.4 ^a,b,c,d^
H7	4.4	20	2	-	172.2 ± 0.4 ^a^	0.10 ± 0.01 ^a^	−40.4 ± 1.5 ^a,b,c^
H8	4.4	20	2	1	210.2 ± 3.2 ^c^	0.12 ± 0.02 ^a,b^	−47.4 ± 3.2 ^d^
H9	4.4	20	2	0.5	182.3 ± 4.4 ^a,b^	0.11 ± 0.04 ^a^	−46.4 ± 1.8 ^c,d^

**Table 3:** Korsmeyer–Peppas Model parameters values for EO release in water and in three Food simulants: FS_A, ethanol at 10%; FS_B, acetic acid at 3%; FS_C, ethanol 20%.

Medium	Korsmeyer-Peppas Parameters					
	PHB NPs			PHB-HHx NPs		
	*R^2^*	*K*_1_	*n*	*R* ^2^	*K*_1_	*n*
FS_A	0.90	19.96	0.31	0.91	19.57	0.33
FS_B	0.86	11.97	0.23	0.91	11.75	0.33
FS_C	0.98	10.58	0.57	0.91	25.58	0.35
Water	0.90	8.89	0.17	0.89	17.03	0.28

**Table 4:** MIC and MBC of free and encapsulated EO against M. luteus. Samples correspond to NPs obtained in trial S13 and H8 for PHB and PHB-HHx, respectively. Values in brackets refer to the concentration of encapsulated EO, based on the experimentally determined Loading capacity (51% and 45% for PHB and PHB-HHx, respectively).

	MIC (mg mL^−1^)	MBC (mg mL^−1^)
EO	0.2	0.3
EO loaded PHB-NPs	0.5 (EO, 0.25)	1 (EO, 0.51)
EO loaded PHB-HHx-NPs	0.25 (EO, 0.11)	0.4 (EO, 0.18)

## Data Availability

Data sharing not applicable.

## Supplementary Materials

The following are available online at https://www.mdpi.com/article/10.3390/polym14010166/s1, Figure S1: Size distribution by Intensity of PHB-based NPs (Sample S3), Figure S2: Size distribution by Intensity of PHB-based NPs (Sample S10), Figure S3: X-ray powder diffraction profiles of the neat PHB (A) and neat PHB-HHx (B), Figure S4: DSC thermograms, acquired at 10 °C/min, of the neat PHB (A) and neat PHB-HHx (B).
