# Supplementary material for: Polyhydroxyalkanoates-Based Nanoparticles as Essential Oil Carriers
Source: Polymers (Basel). 2022 Jan 1;14(1):166. doi: 10.3390/polym14010166 (PMC8747694; doi:10.3390/polym14010166)
Supplement: Supplementary file 1 [file polymers-14-00166-s001.zip › polymers-1506619-supplementary.pdf]

Supplementary materials

Polyhydroxyalkanoates-based nanoparticles as essential oil carriers

Iolanda Corrado [1], Rocco Di Girolamo [1], Carlos Regalado González [2] and Cinzia Pezzella [3,*]

[1] Department of Chemical Sciences, University of Naples Federico II, Via Cinthia, 4 -80126 Napoli, Italy; iolanda.corrado@unina.it (I.C.); rocco.digirolamo@unina.it (R.DG)
[2] Facultad de Química, Departamento de Investigación y Posgrado en Alimentos, Universidad Autónoma de Querétaro, Querétaro 76010, Mexico; regcarlos@gmail.com
[3] Department of Agricultural Sciences, University of Naples Federico II, Via Università, 100 80055 Portici (NA), Italy; cpezzella@unina.it
* Correspondence: cpezzella@unina.it ;

Figure S1 Size distribution by Intensity of PHB based NPs (Sample S3).

Figure S2 Size distribution by Intensity of PHB based NPs (Sample S10).

Figure S3 X-ray powder diffraction profiles of the neat PHB (A) and neat PHB-HHx (B).

Figure S4 DSC thermograms, acquired at 10°C/min, of the neat PHB (A) and neat PHB-HHx (B).

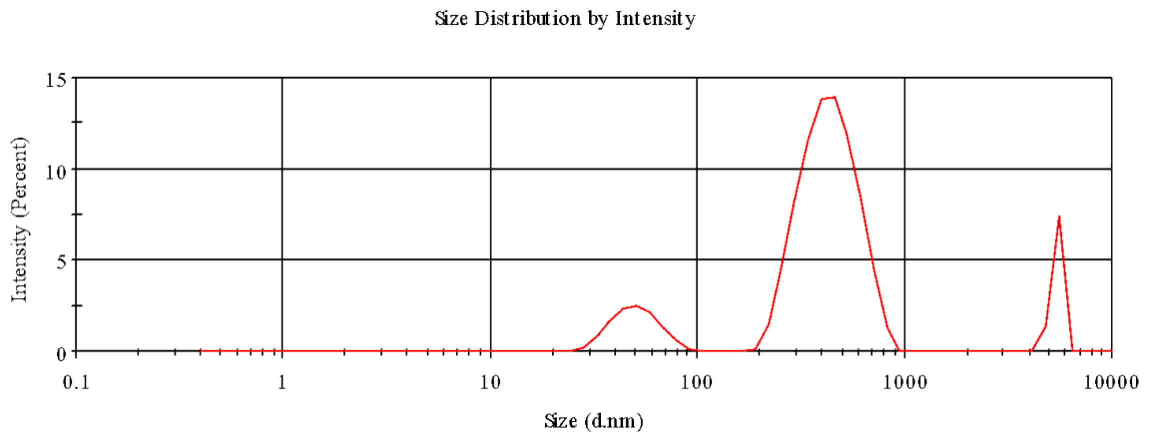

Figure S1. Size distribution by Intensity of PHB based NPs (Sample S3).

The graph results from Z-seizer analysis of sample S3, reporting the size distribution as a plot of the % intensity of light scattered by particles versus various size classes expressed in nm (scale is logarithmically spaced).

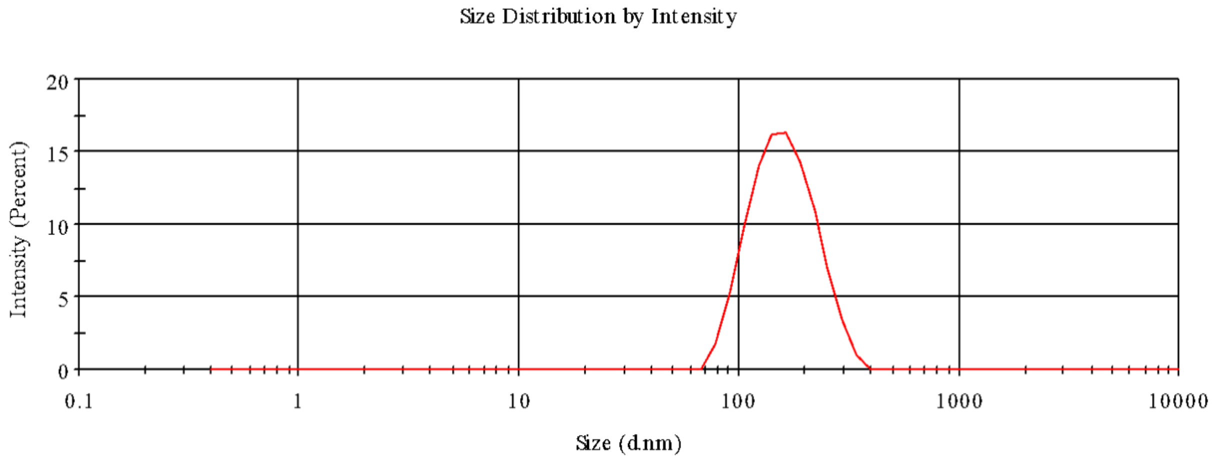

Figure S2. Size distribution by Intensity of PHB based NPs (Sample S10).

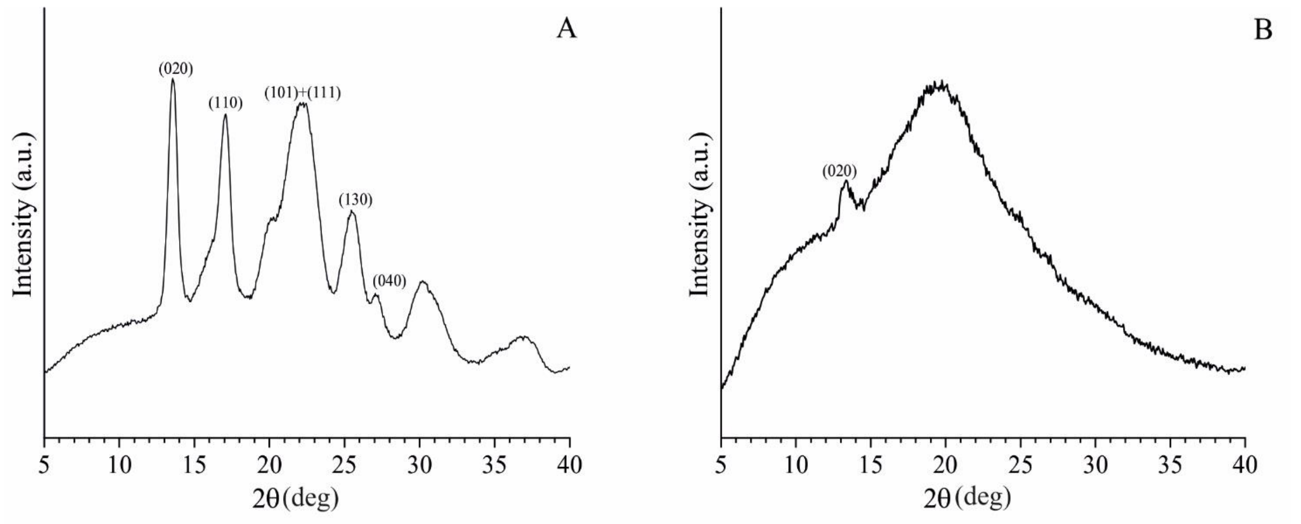

Figure S3. X-ray powder diffraction profiles of the neat PHB (A) and neat PHB-HHx (B).

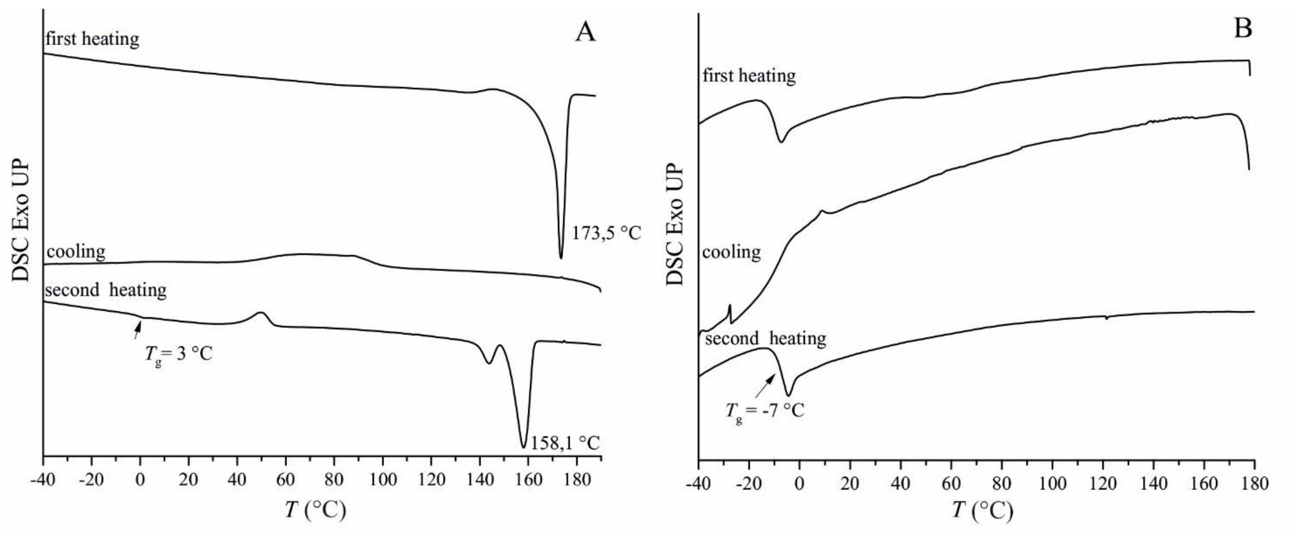

Figure S4. DSC thermograms, acquired at 10°C/min, of the neat PHB (A) and neat PHB-HHx (B).
